# The expansion of heterochromatin blocks in rye reflects the co-amplification of tandem repeats and adjacent transposable elements

**DOI:** 10.1186/s12864-016-2667-5

**Published:** 2016-05-04

**Authors:** E. V. Evtushenko, V. G. Levitsky, E. A. Elisafenko, K. V. Gunbin, A. I. Belousov, J. Šafář, J. Doležel, A. V. Vershinin

**Affiliations:** Institute of Molecular and Cellular Biology, Siberian Branch of the RAS, Novosibirsk, Russia; Institute of Cytology and Genetics, Siberian Branch of the RAS, Novosibirsk, Russia; Novosibirsk State University, Novosibirsk, Russia; Institute of Experimental Botany, Centre of the Region Haná for Biotechnological and Agricultural Research, Olomouc, Czech Republic

**Keywords:** Tandem repeats, Transposable elements, Subtelomeric heterochromatin, Rye, *Secale cereale*, 1RS BAC library, 454 sequences, TE–tandem junctions, DNA motifs

## Abstract

**Background:**

A prominent and distinctive feature of the rye (*Secale cereale*) chromosomes is the presence of massive blocks of subtelomeric heterochromatin, the size of which is correlated with the copy number of tandem arrays. The rapidity with which these regions have formed over the period of speciation remains unexplained.

**Results:**

Using a BAC library created from the short arm telosome of rye chromosome 1R we uncovered numerous arrays of the pSc200 and pSc250 tandem repeat families which are concentrated in subtelomeric heterochromatin and identified the adjacent DNA sequences. The arrays show significant heterogeneity in monomer organization. 454 reads were used to gain a representation of the expansion of these tandem repeats across the whole rye genome. The presence of multiple, relatively short monomer arrays, coupled with the mainly star-like topology of the monomer phylogenetic trees, was taken as indicative of a rapid expansion of the pSc200 and pSc250 arrays. The evolution of subtelomeric heterochromatin appears to have included a significant contribution of illegitimate recombination. The composition of transposable elements (TEs) within the regions flanking the pSc200 and pSc250 arrays differed markedly from that in the genome a whole. Solo-LTRs were strongly enriched, suggestive of a history of active ectopic exchange. Several DNA motifs were over-represented within the LTR sequences.

**Conclusion:**

The large blocks of subtelomeric heterochromatin have arisen from the combined activity of TEs and the expansion of the tandem repeats. The expansion was likely based on a highly complex network of recombination mechanisms.

**Electronic supplementary material:**

The online version of this article (doi:10.1186/s12864-016-2667-5) contains supplementary material, which is available to authorized users.

## Background

Cultivated rye (*Secale cereale, 2n = 2x* = 14) is, after wheat and barley, a major temperate cereal species. Its large nuclear genome of around 8 Gb/1C [1] exceeds that of the average angiosperm (5.6 Gb) [2]. A distinctive feature of the karyotype is that each chromosome arm harbors one or more large blocks of subtelomeric heterochromatin [3], which is not the case in either wheat or barley chromosomes [4, 5]. Within the genus *Secale*, nuclear genome size varies by some 15 % [1], consistent with the variation in the size of the terminal heterochromatic blocks [6]. It would appear, therefore, that the expansion of subtelomeric heterochromatin is fundamental to the determination of genome size in the genus *Secale*.

The rye genome as a whole comprises >90 % repetitive DNA [7]. Eukaryotic tandemly arranged repetitive sequences are typically based on monomers longer than 100 nt [8]; transposable elements (TEs) represent the other major class of multi-copy sequence. In rye, unlike in human and many plant species [9], tandem repeats are concentrated in the subtelomeric region rather than around the centromere [10–12]. Notably, the size of heterochromatic blocks in different rye species correlates with the copy number of tandem DNA families [10]. Molecular organization of the three most abundant of them, pSc119.2, pSc200, and pSc250, was characterized previously [11–13]. They are composed of monomers 118, 379, and 571 bp long, correspondingly, and each family constitutes several percent of the rye nuclear genome [12]. Fluorescence *in situ* hybridization (FISH) experiments have suggested that the pSc200 and pSc250 blocks coincide close to the telomere, while some pSc119.2 copies are located at interstitial sites. The pSc119.2 sequence is also represented in a number of other cereal genomes, but pSc200 and pSc250 are largely rye-specific [14]. Another tandem repeat family (*Tai*I), which is present in many Triticeae species [15], is present on two of the seven rye chromosomes, including the short arm of chromosome 1R (1RS) [16].

Despite a wealth of information regarding the monomers’ size and sequence, their higher order structure remains obscure, as do the mechanisms underlying their amplification. Direct sequencing is hampered by their repetitive nature [17]. Hence their long-range organization, mutual arrangement within arrays, and molecular features of flanking regions between tandem arrays and neighboring non-tandem DNA remain poorly explored (except perhaps for humans). These obstacles can be overcome by approaches allowing one to combine long- and short-range sequence information. These include the construction of BAC (Bacterial Artificial Chromosome) libraries with individual BAC clones containing long (~200 kb) stretches of DNA, and chromosome isolation [18], which enables analysis of DNA organization in individual chromosomes. In a previous work, a BAC library was constructed from the 1RS arm, which was purified by flow sorting from a wheat-rye ditelosomic 1RS addition line [18]. At least 84 % of 1RS arm was found by BAC-end sequencing and Roche 454 sequencing to consist of repetitive DNA and more than 5 % of the 1RS DNA was occupied by 3121 genes [19, 20].

With the exception of 1RS arm, DNA composition, molecular structure of rye genome received little attention, as compared to the genomes of the closest relatives, wheat and barley. It was not until recently that a virtual linear gene order model encompassing over 22,000 out of 31,008 detected rye genes has been established using a combination of high-throughput transcript mapping, 454 sequencing DNA of flow-sorted rye chromosomes, and synteny information of sequenced model grass genomes [21]. Nonetheless, large-scale molecular organization of subtelomeric heterochromatin in rye chromosomes remains completely unknown.

Here we address a number of questions, such as whether major families of tandem repeats are present as a single or multiple arrays in chromosome arms, what is the organization of monomers belonging to each family in different arrays, whether arrays composed by different family members are immediately adjacent to each other or are separated by some DNA sequences, what is the nature of non-tandemly repeated DNA flanking the arrays, and whether it shows any peculiar sequence features. This information may shed light on the molecular organization of heterochromatic regions as well as on the expansion mechanisms of tandem repeat families in the genome of cultivated rye, *S.cereale*.

## Results

### The pSc200 and pSc250 repeats are present in rye chromosomes as multiple arrays

**Fig. 1:**
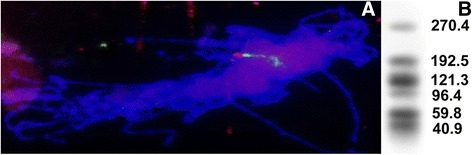
Multiple arrays of tandemly repeated families are present on the short arm of the rye chromosome 1R (1RS). **a** FISH image of early meiotic prophase chromosomes of CS/1RS, showing the location of pSc200 (fluorescing green) and pSc250 (red) sequences. **b** Southern hybridization profiles of CS/1RS probed with pSc250. The size of hybridization fragments is shown in kb

Multicopy tandem repeat families pSc200 and pSc250 appear as strong and homogeneous signals upon FISH with mitotic metaphase chromosomes [12]. Meiotic chromosomes are less condensed at early prophase stage, than metaphase chromosomes, thereby providing higher resolution. FISH analysis of meiotic prophase chromosomes sampled from the wheat-rye monotelosomic addition line harboring 1RS (CS/1RS) showed that both the pSc250 (fluorescing red in Fig. 1a), and pSc200 (green) sequences are present in extensive, non-overlapping domains on the rye chromosome arm. When the 1RS BAC library was hybridized with pSc200 and pSc250, not one of the nearly 67,000 clones analysed hybridized with both probes, confirming that the two array types are not intermingled.

The FISH signal intensity varied along the length of the rye arm (Fig. 1a), in line with the idea that each family of tandem arrays is present as multiple interspaced arrays. Furthermore, this is confirmed by the results of pulsed field gel electrophoresis (PFGE) when *Bst*XI-digested high molecular weight CS/1RS DNA was subjected PFGE and hybridized with pSc250. Six hybridizing fragments were revealed, ranging in length from 40 kb to 270 kb (Fig. 1b). The observed variation in hybridization intensity, taken to indicate that at least some of the arrays harbored non-pSc250 sequence, complicated the quantification of copy number of arrays in 1RS. A restriction analysis, followed by sequencing of the two 1RS BAC clones 12I5 and 122 F3, suggested that both harbored uninterrupted pSc250 arrays (of length, respectively, 38 kb and 57 kb) flanked on either side by non-array sequence. The conclusion is that the 1RS arm harbors several tandem arrays of pSc200 and pSc250 monomers.

### Heterogeneity of tandem array organization

**Fig. 2:**
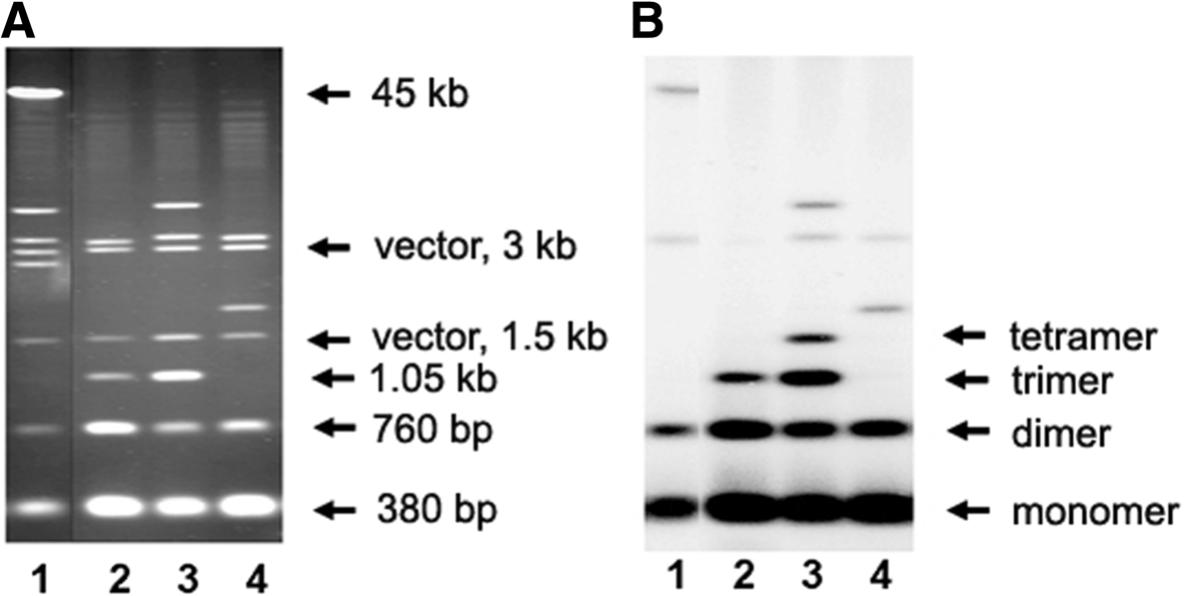
PFGE separation of *Pst*I-restricted BAC inserts harboring pSc200 arrays. **a** Ethidium bromide stained gel, **b** Southern hybridization probed with pSc200. Lane 1: BAC clone 126C20, lane 2: 119C15, lane 3: 119 M22, lane 4: 114I10

Some families of tandemly repeated DNA sequences, such as human α-satellite DNA are known to form higher order repeat (HOR) units that may contain variable numbers of basic repeats (multimers) having highly similar sequences of monomers [22, 23]. HORs were demonstrated to form in the centers of alpha-satellite DNA arrays, with monomeric DNA locating towards their edges [22]. The pSc200 and pSc250 tandem arrays formed higher order multimers, as shown by the ladder-like patterns seen in the Southern hybridization profiles. *Pst*I-digested BACs 126С20 and 114I10 included pSc200 hybridizing fragments consistent with the presence of both monomers and dimers (Fig. 2), whereas the profile of 119С15 suggested the presence of trimers аnd that of 119М22 trimers and tetramers. A *Hin*dIII digestion of clone 114I10 produced a profile consistent with the presence of tetramers and hexamers, while clone 119C15 harbored octamers (data not shown). pSc250 multimers (up to hexamers) have previously been identified [16]. Thus, tandem arrays are organized into specific multimeric units within one chromosome arm. The maximum size of these multimers appears to be ~3 kb for pSc200 (octamer) and ~3.5 kb for pSc250 (hexamer).

When analysing BAC clones from the 1RS library we identified five partially overlapping clones with inserts of different size, each containing pSc200, *Tai*I and pSc119.2 arrays (shown in Additional file 1, central part). Identical non-tandem DNA was sequenced in all five clones indicating that they originated from the same genomic region. Differences in the hybridization patterns produced by these BACs allowed us to accurately position multimeric units within the tandem arrays. For example, unlike the pSc200 monomers and dimers, the tetramers of pSc200 are absent in BAC 130H7 (Additional file 1, right side, line 2). In contrast, other BACs with longer segments of pSc200 array contain tetramers. As inferred from the hybridization patterns obtained with both pSc119.2 and pSc200 (Additional file 1, left and right sides), pSc200 monomers and dimers, and similarly pSc119.2 trimers and tetramers, tended to lie at the ends of the arrays, whereas the higher order multimers were positioned more centrally.

In order to establish the degree of identity between consecutive monomers within a single array, we sequenced eleven full-length pSc200 monomers (total length 5,700 bp) from ВАС119С15 (Тable 1) by creating nested unidirectional deletions using *Exo*III [24]. Monomers from this region fell into two groups; the first with 96 % identity to the first monomer in the contig (monomer fr47-1), and the second with 89 % identity (Table 1). The monomers from these groups alternated in the array, as pairs suggesting their origin via duplication.

**Table 1:** Percent identity of pSc200 monomers present in BAC clone 119C15

Sequence of the pSc200 monomers in contig	fr47-1	fr47-2	exo2-1	T79-1	T79-2	exo6-1	exo7-1	T713-1	exo9-1	exo9-2	T7-6
fr47-1	100	89.36	95.77	88.56	95.23	89.04	98.41	88.86	95.49	89.36	95.77
fr47-2		100	90.16	96.55	89.33	97.60	90.43	99.47	89.36	96.82	89.92
exo2-1			100	90.16	98.41	89.84	96.83	89.66	98.41	90.69	98.94
T79-1				100	89.33	96.27	89.63	96.02	89.10	97.61	89.92
T79-2					100	89.01	96.82	88.83	98.94	89.87	99.47
exo6-1						100	90.11	97.07	89.07	97.07	89.60
exo7-1							100	89.92	97.08	90.43	97.35
T713-1								100	88.86	96.29	89.39
exo9-1									100	89.63	99.73
exo9-2										100	90.45
Т7-6											100

Fragment of the pSc200 array from DNA BAC199C15 was sub-cloned in a plasmid vector pGem-5Zf(+). Then a series of deletion clones was obtained according to [24] and their inserts were sequenced and assembled into a contig encompassing 11 full-length monomers of pSc200

### Phylogenetic relationships between the pSc200 and pSc250 copies

**Fig. 3:**
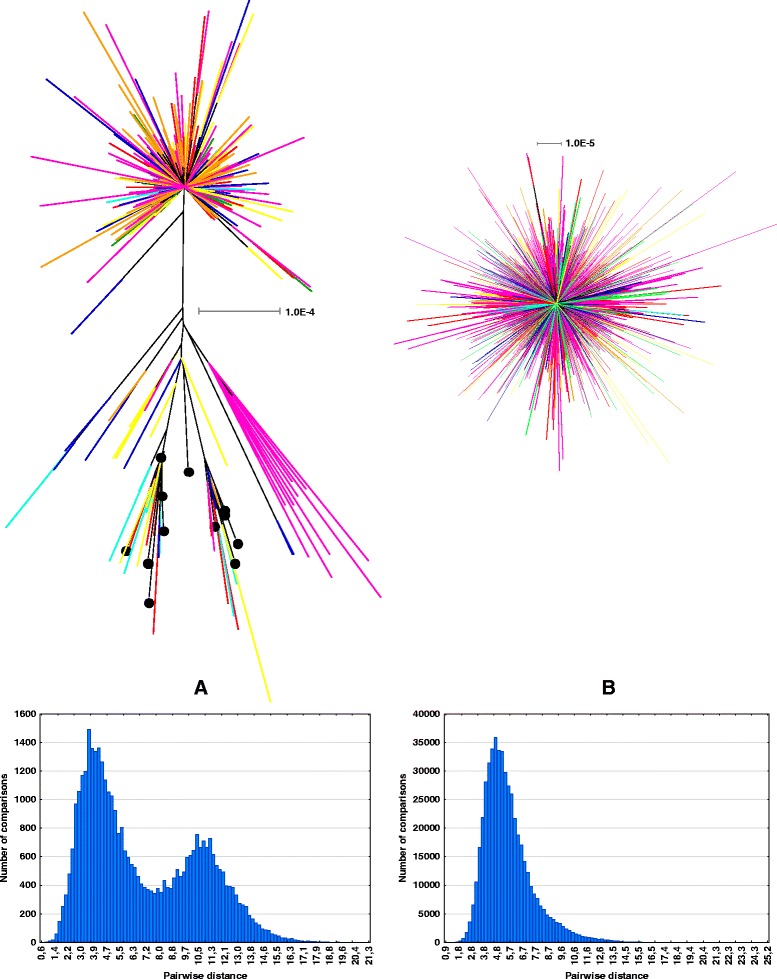
Phylogeny of (**a**) pSc200 and (**b**) pSc250 monomers present on each of the seven rye chromosomes. The chromosome-specific 454 libraries obtained by Martis et al. (2013) were used to reconstruct phylogenetic networks and assign pSc200 and pSc250 monomers to each of the seven rye chromosomes (1R and 1RS chromosomes, red color: ERX140512 ERX140519 libraries; 2R chromosome, orange: ERX140513 library; 3R and 3RS chromosomes, yellow: ERX140514 and ERX140520 libraries; 4R chromosome, green: ERX140515 library; 5R chromosome, cyan: ERX140516 library; 6R chromosome, blue: ERX140517 library; 7R and 7RS chromosomes, violet: ERX140518 and ERX140521 libraries). The phylogenetic trees shown represent galled phylogenetic networks generated by Dendroscope v3.2.8 software based on the trees obtained by maximum likelihood method. Black circles at branch ends refer to sequenced pSc200 monomers present in the ВАС clone 119С15 (see Table 1). The scale bar corresponds to the weighted evolutionary distance (GTR nucleotide substitution model) and indicates the weighted number of substitutions per alignment site. The two histograms depicting the distribution of pairwise distances are shown: the *x-*axis plots the sequence pairwise distance (=100 - % of sequence identity) while the *y-*axis plots the occurrence frequency

Two sets of reads were extracted from the 454 dataset [21]: one containing pSc200 (Additional file 2) and the other containing pSc250 (Additional file 3) monomer sequences (see Methods for details). The unrooted maximum likelihood phylogenetic network constructed for the pSc200 monomers present on each rye chromosome comprised two major clades (Fig. 3a), consistent with a history of at least two time-separated bursts of amplification*.* The larger clade exhibited a star-like topology while the other was branched. The pSc200 sequences present on chromosomes 5R and 7R separated into two recognizably distinct sub-clades. These are the only rye chromosomes which have retained fragments from the ancestral Triticeae chromosome a6 [21]. All of the pSc200 monomers analysed displayed a bimodal distribution of pair-wise genetic distances (Fig. 3a). The first peak in this distribution accounted for intra-clade nucleotide diversity (4 % on average), whereas the second reflected inter-clade diversity (11 % on average). Two groups of monomers present in clone 119С15, and organized as alternate dimers, mapped to distinct branches of the second clade (Fig. 3a).

The pSc250 sequences formed a single clade with a star-like topology (Fig. 3b), which arose from the overall high level of sequence identity (85–98 %) between the monomers. Such a situation probably reflects a relatively constant amplification rate over time. Differences in the topology of phylogenetic networks derived for the pSc200 and pSc250 monomers are consistent with their distinct evolutionary ages: the former originated some 30 My before the latter, allowing ample time for sufficient sequence divergence to have occurred to generate branching in the pSc200 second clade.

### The nature of the sequences flanking the arrays of tandemly arranged monomers in BAC clones

**Fig. 4:**
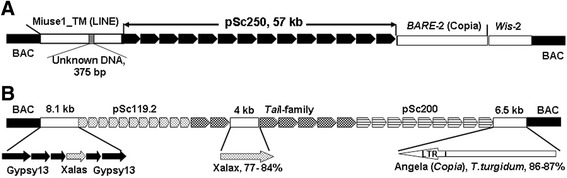
The structure of BAC clones (**a**) 122 F3 and (**b**) 84C15. White rectangles: non-array sequence, black rectangles: vector DNA

To uncover the nature of DNA surrounding tandem arrays of monomers, we sequenced non-tandem (non-array) DNA from six pSc250- and five pSc200-containing BAC clones as well as from three segments of clone 84C15 (Fig. 4) (These sequences have been deposited in GenBank under accession numbers KT724931-48). A screen against the RepeatMasker and TREP databases identified known repetitive elements. The non-array sequences comprised mostly fragments in the size range several tens to several thousands of bp, and shared homology with various families of *Gypsy*-like and *Copia*-like LTR retrotransposons. Short fragments of two LINE elements were also found. No full length TEs were observed. Illustrative examples of the nature of the non-array sequence are given in Fig. 4. In clone 122 F3, the pSc250 tandem array was bordered on the left by sequences which were, respectively, 65.3 and 66.9 % homologous to the central part of the *Miuse* LINE and separated by a 375 nt stretch of anonymous sequence. On the right side, the array was bordered by two *Сopia*-like sequences, one 81.1 % homologous to the *BARE*-2 element and the other 90.5 % homologous to the WIS-2 element (Fig. 4а). Given that *BARE*-2 is a chimera between *BARE*-1 and *WIS*-2 [25], the likelihood is that sequences adjacent to the arrays must have undergone multiple rounds of recombination. The structure of the non-array sequence present in clone 84С15 is consistent with this conclusion (Fig. 4b). Here, the non-array sequence on the right side corresponded to a copy of a *Copia*-like member of *Angela* family, although oriented in the antisense direction, while the left part comprised three copies of the *Gypsy-13_TA-I* element interrupted by two short sequences (not shown), one of which was a simple repeat, and the other sharing homology with the *Laura* retrotransposon. This region was followed by a 1,100 nt sequence homologous (82.5 %) to that of the *Xalas* retrotransposon (TREP1573) and two more regions homologous to *Gypsy-13_TA-I*. Most of the non-array sequence in the central part of the BAC shared homology with the *Xalax* retrotransposon (TREP3344) and was embedded in a tandem array of *Tai*I monomers. This heterogeneous mosaic organization was also characteristic of the pSc200 and pSc250 monomers. Several examples reported in the literature have documented arrays of tandem repeats comprising segments of various TEs, predominantly LTRs [26, 27] but such a scenario of the pSc200 and pSc250 origin from known TEs appears unlikely.

### Characterization of the rye genome composition

The availability of 454 reads derived from each rye chromosome [21] has provided an opportunity to characterize the sequence composition of the rye genome more globally, and to extend the analysis of sequences flanking the tandem arrays in single BAC clones to a genome-wide level. After trimming the adapters and applying quality filtering, the retained set of 14.66 million 454 reads covered about 7 Gbp (mean length: 478 nt). After a filtration step based on RepeatMasker and TREP, two subsets were generated – one containing pSc200 and flanking DNA (314 reads) and the other (494 reads) pSc250 and flanking DNA. These were considered to represent the junction regions (sets “junction”) between non-array sequence and the tandem arrays.

Table 2 shows the frequency of individual classes of TE sequence present in the non-array sequence, of which almost 71 % was unambiguously identified. Among the class II TEs (transposons), there was a notable enrichment of the *CACTA* superfamily (encompassing *Jorge, Pavel, Clifford, Tat1* and other families*)*, a feature which has also been noted in other Triticeae genomes [28, 29]. Other transposon families were poorly represented (just 1.5 % of the sequence). An analysis of the reads showed that most of the repetitive DNA in the rye genome was represented by class I TEs, particularly *Gypsy* LTR retrotransposons. The observed frequency of *Gypsy* sequence was 5.2 fold that of *Copia,* consistent with the estimated 5.8 fold ratio associated with the 1RS arm [20]. Good agreement between genome-wide and 1RS arm frequencies was also observed for all repeated DNA elements, major transposon superfamily CACTA and tandem repeats [19, 20]. Significant differences in the abundance of the major retrotransposon families were noted in the genome as a whole compared to that found in the vicinity of the tandem arrays. *Gypsy*-like TEs were less frequently associated with tandem arrays (Тable 2), but were more common in the flanking sequence of the pSc200 arrays than that of the pSc250 ones. The occurrence of *Copia*-like TEs increased in the vicinity of the pSc200 arrays. Most importantly, there was substantial enrichment of solo LTRs around the pSc200 arrays (12.8 %) and pSc250 arrays (23.0 %) which exceeds the frequency of all the *Copia*-like elements.

**Table 2:** Sequence composition of genome-wide 454 reads and of the sequences adjacent to pSc200 and pSc250 arrays

	All reads		Reads with junctions of pSc200		Reads with junctions of pSc250	
Type of sequence	Cumulative length, bp	Proportion to cumulative length, %	Cumulative length of non-tandem DNA, bp	Proportion, %	Cumulative length of non-tandem DNA, bp	Proportion, %
Class I TE						
Ty3/Gypsy-like	4 142 315 628	50.84	36542	35.68	65786	40.62
Ty1/Copia-like	799 492 074	9.81	14322	13.99	14677	9.06
solo-LTR	60 362 185	0.74	13119	12.81	37289	23.02
LINE	57 651 280	0.71	483	0.47	1212	0.75
SINE	1 201 224	0.02	0	0.00	0	0.00
Class II TE						
CACTA	454679975	5.58	5544	5.41	5621	3.47
EnSpm	18661838	0.23	984	0.96	23	0.01
Harbinger	20519563	0.25	0	0.00	0	0.00
Mariner	24069442	0.30	370	0.36	147	0.09
Hat	12376913	0.15	0	0.00	268	0.17
Helitron	3779908	0.05	0	0.00	0	0.00
Others	37550591	0.46	124	0.12	307	0.19
Simple repeats, low complexity	27 883 202	0.34	259	0.25	383	0.24
rDNA	8 782 174	0.11	0	0.00	396	0.24
Tandem repeats	16 953 966	0.21	185	0.18	2420	1.49
Unclassified (unknown)	48 627 218	0.60	2348	2.29	2996	1.85

We computed DNA composition of all reads and compared with that in non-tandem DNA adjacent to the pSc200 and pSc250 tandem arrays. Length of all repeats was defined according to annotations that we got in output files of RepeatMasker tool (see “Methods”). Columns “Proportion” denote the ration of the cumulative length of the given non-tandem DNA to the cumulative length of all reads

### The vicinity of tandem arrays is populated by certain TE families

**Fig. 5:**
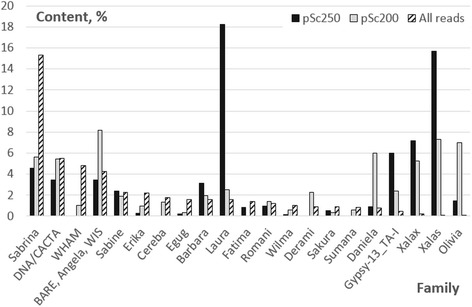
The genome-wide and array-flanking sequence (junction sites) abundance of various TE families. Only those TE families exhibiting a large disparity in abundance are shown.

According to TREP, the vast majority of the solo-LTRs belong to the *Xalax* or *Xalas* groups of TE with minor contribution of *Ginger* element (0.3 % for pSc200 flanks, and 0.12 % for pSc250). Further analysis was conducted in order to identify the proportions of different TE families present in the genome as a whole, in the junctions between pSc200 or pSc250 arrays and in the neighboring non-array sequence. The total length of a given TE’s sequence across all reads was calculated, and then normalized by overall reads’ length. In all, some 400 TE families were identified, of which 26 most abundant families represented 50.13 % of the identified TEs. The abundance in the genome of the *Gypsy* family *Sabrina* was particularly high (15.5 %), with the next most abundant families ranging in occurrence from 2 to 5 % (Fig. 5).

*Gypsy-13_TA-I*, *Xalax/Xalas* and *Olivia* are all relatively rare in the rye genome (0.5, 0.3 and 0.05 %, respectively), but their abundance was noticeably higher in the flanking DNA of the pSc200 and pSc250 arrays, particularly in the case of *Xalax/Xalas*. The *Xalax/Xalas* supergroup cannot be classified as a single family given the level of sequence diversity present, if one follows the rules of the unified classification [30], rather it should be considered as two groups. *Xalas* elements are very homogeneous, while homology between *Xalax* and *Xalas* is restricted to relatively short sequence blocks (shown in Additional file 4). Thus, here, *Xalax* and *Xalas* were treated as independent TEs; enrichment around the pSc250 arrays was predominantly composed of *Xalas* sequence (Fig. 5).

Several other families of TEs show behavior similar to that of *Xalax/Xalas* around the pSc200 and pSc250 arrays (Fig. 5). While *Gypsy-13_TA-I* was more highly enriched around the pSc250 than around the pSc200 arrays, the opposite was the case for *Olivia*. There was a pronounced difference between the genome-wide and tandem-array associated frequency of some other TEs. For instance, *Daniela* was enriched 7.5 fold in the vicinity of the pSc200 arrays, and *Laura* occurred 11.5 fold more frequently in regions adjacent to pSc250 (Fig. 5). At the same time, sequences around tandem arrays are depleted with respect to *Sabrina*, the most abundant TE in the genome as a whole. The differences between genome-average and local enrichment values for some other TE families, such as *CACTA* and *Sabine*, are small. Several TEs were virtually absent from the tandem array flanking sequence, namely *Cereba, Derami, Sumana* around pSc250 and *Fatima* around pSc200. Thus, our analysis clearly shows that the local sequence composition around tandem arrays differs dramatically from the genome average, and displays several peculiar features.

### Structural features of the TE/tandem repeat junctions

**Fig. 6:**
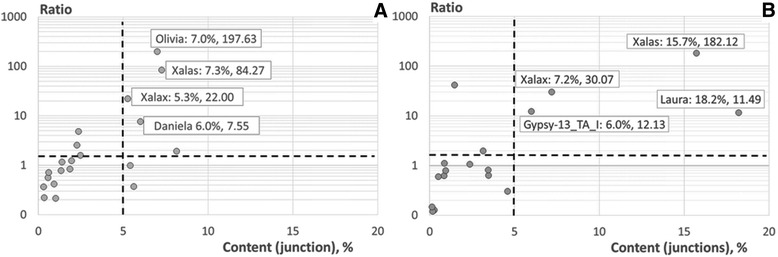
The most highly enriched TE families localizing in the vicinity of pSc200 and pSc250 arrays. The *x-*axis plots the percent of TE/array junctions harboring a particular TE family, while the *y-*axis plots TE enrichment relative to the genome average at the junction site. Dashed lines delimit the areas on the plot used for TE family selection. Selected TE families are indicated and are followed by their x and y values. ТЕs located in the vicinity of (**a**) pSc200, (**b**) pSc250

A *de novo* search for DNA motifs in TE/tandem repeat junctions was conducted to determine whether there was any uniqueness with respect to the nucleotide composition present in these regions. Twelve top-scoring motifs (8–12 nt long) from the output of *de novo* search tool Homer (see Methods) were selected as the most enriched in the neighborhood of each family (Table 3). None of these motifs featured in the vicinity of both tandem array families. A search for motif enrichment within junction sequences containing the commonest junction-associated TEs was made by applying two criteria: first that the TE was present in at least 5 % of all junctions and second that the TE-containing junction displayed an at least two fold enrichment for the motif. Only eight TEs satisfied both criteria (Fig. 6): four in the vicinity of pSc200 (*Daniela, Olivia, Xalas* and *Xalax*) and four in the vicinity of pSc250 (*Laura, Xalas, Xalax* and *Gypsy-13_TA-I*). As is the case for *Xalas* and *Xalax*, *Daniela* and *Olivia* share extensive regions of homology within their LTRs (Additional file 4). Among various associations between the twelve motifs and the top pSc200/250 array-enriched TEs, the strongest involved *Daniela* and *Olivia* around pSc200, and *Laura* and *Xalas* around pSc250 (Table 3). Most of the motifs were represented in at least one of the TE families. In the vicinity of the pSc200 arrays, five motifs were identified in *Olivia* (ten motifs in total) and *Daniela* (five motifs). In the non-array sequences around pSc250, six motifs were present in *Laura* and eight in *Xalas*: five of these were in common. Some closely related sequences were also detected within the pSc200 and pSc250 arrays. For example, pSc200 harbored CACAGGATCA (*P* < 4.1e^−5^ with respect to motif 6) and CAACGCCTATG (*P* < 2.5e^−5^, motif 10) (Table 3А), while pSc250 harbored GTAACCTGGCC (*P* < 4.9e^−6^, motif 10) (Table 3В).

**Table 3:** Enrichment estimates (*t-*test) for top-scoring motifs

А. Top-scoring motifs in TE - pSc200 junctions, Logo			
Motifs		t-test, P*	
		Olivia	Daniela
1		1.8E-04	
2		1.4E-09	1.3E-03
3		1.4E-09	7.6E-04
4		2.0E-07	3.6E-03
5		5.2E-13	
6			
7		9.5E-04	
8			
9		1.8E-09	4.8E-06
10		7.8E-06	
11		1.7E-08	1.4E-04
12		1.7E-05	
В. Top-scoring motifs in TE - pSc250 junctions, Logo			
Motifs		t-test, P*	
		Laura	Xalas
1			
2			3.8E-05
3			1.2E-04
4			
5			
6		1.3E-04	
7		1.9E-03	1.4E-03
8		1.0E-07	4.0E-03
9		8.8E-04	4.0E-03
10		4.0E-05	1.1E-09
11			2.6E-06
12		1.4E-09	1.6E-03

Top-scoring motifs present in the (A) TE/pSc200 junctions, (В) TE/pSc250 junctions, Logo. *significance of enrichment was estimated by Fisher’s t-test as described in “Methods” the only statistically significant values are shown

Next, we explored whether there is any regularity in the localization of TEs in these regions, i.e. whether TEs tend to break at LTRs, or at their central domains, and whether unrelated spacer sequences may be present at the junctions. Additional file 5 illustrates this distribution for the four chosen TE families. The spacer sequences between the TEs and the arrays were either absent, or at best short (1–10 nt). About 90 % of the junctions between pSc250 and *Laura*/*Xalas* elements fell into this category, as did most of the junctions between pSc200 and *Daniela* (70 %) and *Olivia* (58 %).

At the junction points, most TE copies began with the LTR’s 5′- or 3′-terminal nucleotide, or a nucleotide very close to the terminus (the distribution of distances is shown in Additional file 6). Analysis based on RepeatMasker software showed that half of the *Laura* elements present in the vicinity of pSc250 began with their first or last 1–10 nucleotides. Similarly, 54 % of *Daniela* copies and 68 % of *Olivia* copies in the vicinity of pSc200 began within the first 20 nucleotides of their LTR; as for pSc250, 69 % of adjacent *Xalas* copies began with nucleotides 1–20 of one or other LTR. Mapping the top-scoring motifs against the sequences of *Daniela, Olivia*, *Laura,* and *Xalas* extracted from TREP database revealed the same trend, namely the motif density was the highest within the first 300 bp of their 5′- or 3′- LTRs (data not shown) particularly with respect to motifs 2 and 9 in the *Daniela* and *Olivia* LTRs.

Thus, our analysis of genomic DNA composition and de novo identification of DNA motifs overrepresented in the vicinity of pSc200 and pSc250 sequences uncovered enrichment of these regions with 5′- and 3′-LTRs of *Olivia/Daniela* and *Laura/Xalas* TEs, respectively. These results point to the substantial role of nucleotide context in the formation of DNA flanking tandem repeats, which is likely based on its involvement in the molecular mechanisms taking place during amplification of tandem arrays and associated recombination events.

## Discussion

### Multiple arrays of tandem repeats with distinct higher-order organization are present in the chromosomes of rye

The organization of tandemly repeated sequences is poorly understood, not just in the cereals, but in eukaryotes generally. A notable exception is the human α-satellite, comprising a large array at each centromere which emerged as a paradigm for understanding the genomic organization of other tandem DNA sequences [22, 23, 31–34]. Although these latter arrays are mostly homogeneous, a few chromosomes harbour two or more distinct arrays each defined by different HORs [31, 32]. Here, the application of FISH clarified that the pSc200 and pSc250 arrays located close to the 1RS telomere are organized into discrete domains, a conclusion supported by the Southern hybridization analysis. We believe that this observation can be extrapolated to heterochromatin regions of other rye chromosomes. The evidence for this extrapolation is supported by the size of the set of junction site sequences (314 involving pSc200 and 494 involving pSc250), which number far more than the number of chromosome arms. It is possible that some of these reads arose from non-array sequence embedded in the monomer array, but these cannot be common since none emerged from the sequencing of several 1RS BAC clones. The frequency of direct junctions between pSc200 and pSc250 arrays is very low; only six reads fell into this category, which further reinforces our conclusion that each of these two families has its own, distinct localization domain on the rye chromosomes. Nonetheless, the fact that pSc200 and pSc250 FISH signals display partial overlap is indicative of the close proximity and short junction regions between both domains.

HORs are composed of monomers with nearly identical nucleotide sequence and are found located in the centers of alpha-satellite DNA arrays [22]. In this work we show that in rye, pSc200 and pSc250 sequences form multimeric units with the number of monomers varying from 2 to 8 and that the multimeric units map to the centres of the arrays. Multiplicity of monomers within these multimeric units is specific for each individual array found in 1RS. This may argue in favor of multiple recombination events involving distinct tandem arrays within one chromosome arm, which led to HOR formation. Supportive evidence of active recombination within tandem arrays is provided by the level of sequence divergence (up to 7 %) observed between pSc200 monomers arranged as dimers within a BAC119C15, which implied that two monomers first formed a single unit, which was later amplified as a unit. It has been suggested that, during the evolution of tandem arrays, early duplication events were more frequent than subsequent amplification steps [34]. The dimeric repeat structure is universal for alpha satellite DNA, as it is present across various Old World monkey species [35] and is 15–20 MY old based on the estimated evolutionary divergence of these species [36].

### A comparison of rye tandem repeat families and primate α-satellite DNA

Most of the pSc200 and pSc250 arrays ranged in length from 40–300 kb [13], while the human α-satellite forms much longer arrays of up to 6 Mbp [23]. The two array types are also located in a different part of the chromosome (subtelomere *vs* centromere). The postulated mechanisms for the generation and maintenance of tandem arrays include unequal sister-chromatid exchange, sequence conversion, translocation exchange and transposition [8, 37, 38]. As most α-satellite subsets are chromosome-specific, the within homologs exchange frequency is thought likely to be significantly higher than that occurring between non-homologs [38]. The pSc200 and pSc250 tandem arrays appear to have distinct evolutionary histories. Several pSc200 copies are present in hexaploid wheat and other *Triticeae* species [14, 39], but has also been identified in the more distantly related rice and oat. As a result, it must have arisen at least 45 Mya, when the rice and oat lineages diverged [40], making it more ancient than the human α-satellite, whose presence throughout the primate order dates it to some 35 Mya [38]. The pSc250 sequence is much younger; its appearance as isolated copies in a few *Triticeae* species [14] dates it to 15 Mya. Despite their representation across multiple grass species, the expansion of both families has postdated the divergence of *Secale* from its closest relatives [41]. Thus, both families have been amplified over a much shorter timescale than α-satellite DNA.

The topology of both the pSc200 and pSc250 phylogenies was largely star-like, in contrast to the tree-like form of the human α-satellite phylogeny [33]. With the exception of the chromosome 5R and 7R sub-clades, the chromosomal origin of the pSc200 monomers was heterogeneous. The presence of multiple, relatively short arrays on each rye chromosome, along with a predominantly star-like phylogeny, are consistent with the rapid evolution of these arrays, likely accelerated by illegitimate recombination including interchromosomal recombination events. This model is supported by FISH-identified presence/absence and intensity polymorphisms for both pSc200 and pSc250 between homologs of different cereal rye accessions [41, as well as the readiness with which introgression occurs in *S. cereale x S. montanum* hybrids [42]. Exchange of satellite sequences between chromosomes is not unprecedented and was demonstrated for allopolyploid *Nicotiana* species [43]. The presence of the 5R- and 7R-specific pSc200 sub-clades may be connected with the observation that it is only these chromosomes which still harbor fragments of the ancestral *Triticeae* chromosome a6 [21], but how such ancient DNA segments may have escaped interchromosomal exchanges is not clear.

### The abundance of certain TE families in the vicinity of pSc200 and pSc250 arrays

TEs are responsible for much of the genome enlargement seen in the cereals [29, 44, 45], and their concentration in heterochromatin is well-established. Thus it was expected that TE sequence would be common in the regions flanking the pSc200 and pSc250 arrays. The sequences appeared as a mosaic of incomplete, heterogeneous TEs, likely resulting from nested insertions subjected to subsequent recombination, duplication and indel formation [46, 47]. The analyses of barley and wheat genomic sequence has shown that most TE families are present in relatively low copy numbers and that just 15 families make up at least 50 % of the genome complement [29]. Similarly in rye, the *Sabrina* family constituted an estimated 15.5 % of the nuclear genome. Why particular TE families have been able to expand in a species-specific manner is quite unknown. *Sabrina* was first identified in barley [45] but is widespread in the *Triticeae* [29] including wild species of *Secale* [48]. Although similar to *Gypsy*, it contains an *env*-like gene, the product of which includes predicted transmembrane domain which may aid its horizontal transfer. Notably, in *S. cereale*, *Sabrina* is only seldom seen in subtelomeric regions [48], suggesting that it has not been actively involved in the formation of the prominent heterochromatin blocks.

A striking feature of pSc200 and pSc250 array flanking sequence is that although it has been enriched for TE sequence, the TEs involved were not highly abundant across the genome as a whole. The frequency of solo-LTR elements is particularly notable around pSc250. Ectopic exchanges were likely commonplace in the vicinity of the arrays as this is in line with the predictions of the ectopic exchange model [49]. The solo-LTRs present in the flanking sequence were largely a heterogeneous mixture of *Xalas* and *Xalax*. The former element was first identified in barley [46], and despite its relatively large size (~4 kb), it has not been assigned to any of the LTR-retrotransposon superfamilies, as no coding domain-like sequences have yet been identified. Various representatives of *Xalas/Xalax* share relatively short regions of incomplete homology (rarely >80 %). Thus, these elements cannot be classified as a single family according to the 80-80-80 rule [30]; a similar level of identity applied between the terminal segment of the *Daniela* and *Olivia* LTRs. The major processes likely responsible for the formation of solo-LTRs are unequal crossing over and intrachromosomal ectopic recombination between LTRs of the same or even different elements, when they share the regions of homology. If recombination involves the LTRs of different elements, a range of recombination products may result, potentially leading to chromosome rearrangement [25].

### Multiple recombination mechanisms were likely involved in the expansion of rye tandem repeats and their flanking TEs

Whereas the molecular basis of recombination between tandem repeat has long been an active research topic, little attention has been given to resolving whether the sequences adjacent to the arrays affect the expansion process. The sequences flanking human α-satellite DNA are highly heterogeneous [50] and do not seem to be enriched for TEs [33]. Any recombination event involves the formation of double-strand breaks and their subsequent repair. The latter process is achieved by non-homologous end joining (NHEJ) and homologous recombination (HR) [51]. The present analysis of the array/TE junctions indicated that most of the TE sequence was integrated either directly into the monomers or attached to it *via* a very short (1–10 bp long) spacer, consistent with the NHEJ scenario. Most of the junctions between pSc250 and *Laura/Xalas* and between pSc200 and either *Daniela* or *Olivia* followed this pattern.

A degenerate 13 nt motif has been demonstrated to be associated with ~40 % of human crossover hotspots [52]. Currently, no such clear association between recombination and specific DNA sequence motifs has been established in plants [53]. Nonetheless, the junction regions in rye are clearly enriched with respect to several DNA motifs, some of which may be involved in other known DNA repair mechanisms acting independently of NHEJ and HR [54]. The heterogeneity of DNA motifs found in the TEs, combined with the relatively low level of sequence similarity within the homologous regions of *Xalas*/*Xalax* and *Daniela*/*Olivia*, fit the requirements for microhomology-mediated break-induced replication and gene conversion to function [54]. The length of the motifs identified (8–12 nt) agrees well with a recognition mechanism allowing recombinases to align single-stranded DNA with a homologous duplex (dsDNA). Once a presynaptic complex has engaged a particular 8-nt (or longer) tract of microhomology, it may become exchanged with other region of dsDNA bearing the same microhomology, yet resist exchanges with unrelated sequences [55]. The number of rearrangements induced by microhomology-driven pathways is likely to be higher than is currently thought [54]. The outcomes of these currently under-appreciated repair pathways could include an increased copy number of the sequences being repaired [56]. Consequently, these mechanisms may be a significant contributor to the formation of heterochromatic blocks.

### Shaping the rye genome by tandem repeats

Although the barley and wheat-rye lineages diverged approximately 10–13 Mya [57], and wheat and rye shared a common ancestor only 6–7 Mya [57], the karyotypes of these three species vary drastically with respect to both their size and structure [58], although not with respect to their gene content [21]. It is widely accepted that differences in genome size between closely related species are largely attributable to the quantity of intergenic DNA present, which in turn is heavily influenced by TE copy number and composition. In the case of rye, an increased TE content has not been the sole factor contributing to its genome expansion; in addition there has been a massive amplification of tandemly repeated DNA, based on pSc200 and pSc250 (and other) monomers. This conclusion is supported by the positive correlation between larger heterochromatic blocks and higher content of tandem DNA repeat families in the cultivated rye (*S. cereale*) as compared to wild rye species [10]. The high copy tandem repeats found in barley and wheat, HvRT [59], pSc119.2 [11], dpTa1 [60] and *Tai*I [15], are significantly less abundant than are pSc200 and pSc250 in rye.

The presence of multiple copies of the repeated DNA sequences in each subtelomeric region might be expected to promote pairing between homologous and non-homologous chromosomes. The termini of rye chromosomes are known to play a key role in the initiation of synapsis [61], and since they remain associated for a longer period than other parts of the chromosome, it has been suggested that this explains why the frequency of recombination increases along the centromere-telomere axis [62]. The recombination rate gradient along the centromere-telomere axis is steeper in the wheat close relative *Aegilops speltoides* (the chromosomes of which feature large subtelomeric heterochromatic blocks) than in einkorn wheat (which lacks major blocks) [63].

## Conclusion

Early studies have noted that tandemly repeated DNA can increase in copy number over a relatively short evolutionary time by replication conversion-like events or *via* some other unexplained mechanisms [8]. This phenomenon has recently received further support *via* analysis of the evolutionary fate of various satellite repeats in species from *Nicotiana* section Polydicliae [43]. Significant progress has been made over the past twenty years in understanding the molecular nature of various recombination pathways. Direct involvement of pSc200 DNA in the association of subtelomeric regions of two or more bivalents was demonstrated by FISH [64]. It is highly probable that the heterogeneous composition of pSc200 and pSc250 multimeric units and the localization of multimers to the central part of monomer arrays is a by-product of unequal crossing over and homologous recombination. Gene conversion and ectopic exchanges between homologous and non-homologous chromosomes have promoted the formation of multiple arrays of each repeat family and contributed to a significant enrichment of the flanking sequences with solo LTRs and several TE families. The presence of short microhomology tracts in these elements implies a contribution of other known recombination pathways [54]. Thus, all the above-listed mechanisms may have been involved in creating the bewildering complexity of recombination events that have ultimately resulted in expansion of tandem repeat families pSc200 and pSc250 as well as several TEs in the rye genome.

## Methods

### Plant material and FISH

The plant materials used were the bread wheat cv. Chinese Spring (CS), the cereal rye cv. Imperial and wheat-based ditelosome addition line involving rye chromosome arm 1RS (CS/1RS) [65]. Chinese Spring cultivar is an international standard for wheat research, much as the rye cv Imperial. Spikelets at the appropriate meiotic stage were fixed and prepared for FISH as described elsewhere [66]. FISH was performed according to a protocol optimized for rye meiotic chromosomes [66].

### DNA plug preparation, PFGE and Southern hybridization

High molecular weight DNA was isolated from protoplasts prepared from CS/1RS seedlings [67]. The agarose plugs containing the DNA were loaded into a CHEF-DRIII PFGE system device (Bio-Rad) for PFGE through a 1 % agarose gel. The separated DNA fragments were transferred to a Hybond-N+ membrane, which was then subjected to Southern hybridization at 65 °C following [68], rinsed once at 65 °C in 0.1 M Na_2_HPO_4_, 0.1 % (*w/v*) SDS for 30 min, and then in 0.04 M Na_2_HPO_4_, 0.5 % (*w/v*) SDS for 30 min.

### DNA probes and labeling

For FISH experiments, pSc200 (accession number Z50039.1) and pSc250 (accession number Z50040.1) were labeled with, respectively, digoxigenin-11-dUTP (Roche) and biotin-11-dUTP (Roche) *via* PCR [66]. For Southern hybridization experiments, pSc119.2, *Tai*I, pSc200 and pSc250 were labeled with [α-^32^P]dATP (GE Healthcare, Amersham) either by PCR or by random priming [66].

### Analysis of 1RS-specific BAC library and BAC clone sequencing

Filters with spotted BAC clones from the 1RS-specific BAC library SccImp1RShA [18] were sequentially hybridized with pSc200 and pSc250 probes. Positive clones were selected for preliminary analysis of insert sizes and patterns of restriction fragments. Clones displaying distinct restriction digestion patterns and positive for pSc200 or pSc250 were chosen for finer analyses. Namely, these clones were first subjected to a stability analysis [69], then restriction mapped using either partial digestion with one enzyme or a complete digestion with two [70].

Digested BAC fragments were subjected to pulsed-field gel electrophoresis using CHEF-DR III apparatus (BioRad) in 1 % agarose gel on 0.5хТBЕ at 14 °С. The settings used were as follows: initial switch time - 0.5 s, final switch time – 4 s, voltage - 6 V/cm, running time 10–12 hours, depending on the expected DNA fragment sizes. Following gel electrophoresis, the DNA was transferred onto Hybond-XL membrane (Amersham Biosciences) and subjected to Southern-blotting, as described above.

The primer walking sequencing of the BACs was performed using a ABI PRISM BigDye™ Terminator Cycle Sequencing Ready Reaction kit (Applied BioSystems); the reaction products were separated using an ABI3730xl capillary sequencer. Primers annealing to the ends of the pIndigoBAC-5 vector (used to construct the SccImp1RShA library) were used for the initial walking step. Downstream sequencing reactions used primers designed from *de novo* acquired sequence.

### Subcloning of BAC sequences and sequencing of pSc200 arrays

The portion of the pSc200 array in ВАС clone 119С15 was sequenced by initially digesting it with *Hin*dIII, *Nde*I and *Xba*I. The products were separated by PFGE and probed with pSc200. A hybridizing fragment was gel-eluted using a Min Elute Gel Extraction kit (Qiagen) and the DNA then ligated to *Nde*I/*Spe*I restricted pGem-5Zf(+). The ligation products were transferred into *E. coli* XL10-GOLD (Stratagene) competent cells [70]. A series of deletion clones was obtained by treatment with exonuclease III and SI nuclease (ThermoScientific) [24]. Protruding 5′- and 3′-ends were generated by *Sph*I/*Nco*I digestion (Promega). The resulting 300–400 bp inserts were sequenced and assembled into a contig which comprised 11 full length pSc200 monomers.

### Processing of 454 reads

Chromosome-based rye genome sequence [21] was used to characterize the flanking regions of the pSc200 and pSc250 arrays. Adapter sequences were removed from the reads using tagcleaner 0.12 [71], quality sorting was performed as described in [72], and makeblastdb software [73] was run to create a relevant reads database. Phred quality scores were set as follows: Av(Q) –Z * σ(Q), where Av(Q) and σ(Q) denoted the quality score mean and standard deviation. The Z values were set as 2.6 and 1.6 for the phylogenetic analysis and the analysis of tandem array/non-array junctions, respectively.

### Subsampling of 454 reads for phylogeny construction

Consensus pSc200 and pSc250 sequences, established from archival and BAC clone sequences, were used as blastn and blastcmdsearch queries [73] to extract homologous 454 reads (length thresholds were, respectively, 95 and 80 %). In order to select only distinct monomers with the homology level at most 98 % we run the nucmer and show-coords routines implemented in MUMmer v3.23 [74]. A multiple alignment of the chosen sequences was performed using sate v2.2.7 software [75].

### Phylogenetic analysis of the pSc200 and pSc250 families

Jack-knife analysis [76] was performed to assess the statistical robustness of the predicted phylogeny; this involved the removal of 25 % random aligned regions. This threshold was chosen based on the observation that substitutions/deletions were infrequent and were uniformly distributed. For each jackknifed alignment, the maximum likelihood algorithm implemented in raxml v7.4.2 software [77] and the GTRGAMMA model were used to construct the phylogeny. Based on the set of 500 trees, dendroscope v3.2.8 software [78] was used to build a Galled phylogenetic network of the original set of trees. Pairwise distances were calculated based on the original (all sites intact) alignment of repeats using the distmat program implemented in the EMBOSS v6.3.1 package [79].

### Sampling of the junctions between TEs and tandem repeats

Quantification of the various DNA families was based on the entire set of high-quality 454 rye whole genome DNA reads. Two subsamples of reads (termed “junction”) were compiled: each member’s sequence included a segment homologous to either the pSc200 or the pSc250 sequence (E < e^−06^) using WUBLAST (http://blast.wustl.edu). The non-array portions were oriented and aligned to begin at the TE/tandem repeat junction. Both the total set of reads and the two “junction” subsamples were scanned using RepeatMasker (http://www.repeatmasker.org), TREP (http://wheat.pw.usda.gov/ITMI/Repeats/) software, applying default settings. Finer positioning of the repeats within the reads was achieved using FASTA software [80]. The remaining reads were then filtered to retain those harboring at least 200 nt of non-array sequence.

### Analysis of nucleotide context at the TE/tandem repeat junctions

The “junction” reads from which array sequence had been were removed were trimmed by 80 nt at their 3′-end. We applied the threshold of sequence identity 90 % to non-array DNA in order to analyse only non-redundant junctions. Homer software (http://homer.salk.edu/homer/motif/) was used for the *de novo* identification of enriched motifs. The required set of background sequences was generated by the shuffling of sequences of the test sample. The 12 top-scoring motifs from the output of Homer tool for each of the pSc200/pSc250 families were selected, because this number was sufficient to confirm the hypothesis on the relationship between the most overrepresented motifs and the most abundant TEs. This hypothesis is based on the enrichment of tandem-genomic DNA junction with certain types of TEs. For each top-scoring motif a position weight matrix was obtained from the matrix of nucleotide frequencies using log-odds weights [81]. Each of these matrices was based on the threshold values computed as in [82] applying a *P* value of 5e^−5^. The statistical significance of association between the motif hit occurrence and TE mapping in a read (the “DNA motif – TE” association) was estimated by Fischer’s *t*-test for angular (arcsine square root) transform proportions [83]. The proportions were computed from the ratio between the number of junctions with hits of motif to the total number of junctions. The first proportion *A/(A + C)* referred to the total set of junctions, and the second *B/(B + D)* to the subset of junctions which included a TE, where A through D represented the relevant number of reads (the details are described in Additional file 7). According to Bonferroni’s correction only “DNA motif – TE” associations for which *P* value was <0.00417 (0.05/12) were considered as significant.

### Data availability

The sequence data described are available in GenBank under accession numbers KT724931-48.

## Additional files

Additional file 1: Multimeric repeat units present in the central portion of the arrays. PFGE separation of BAC clones containing pSc119.2, *Tai*I and pSc200 arrays. Southern hybridizations probed with (left panel) pSc119.2 and (right panel) pSc200. Lane 1: BAC clone 84C15, lane 2: 130H7, lane 3: 230C21, lane 4: 230N4, lane 5: 241H2. The central panel illustrates the structure of the BAC clones. Black rectangles: non-array sequences. (PDF 90 kb) Additional file 2: Set of pSc200 family monomers used for reconstruction of phylogenetic network. File can be opened in BioEdit Sequence Alignment Editor. The name of each sequence consists of two parts: the right part from the dot points the chromosome-specific library, the left part from the dot points the read number in library. (TXT 115 kb) Additional file 3: Set of pSc250 family monomers used for reconstruction of phylogenetic network. File can be opened in BioEdit Sequence Alignment Editor. The name of each sequence consists of two parts: the right part from the dot points the chromosome-specific library, the left part from the dot points the read number in library. (TXT 550 kb) Additional file 4: Dot-plot alignment of TE sequences most highly enriched in the TE/tandem array junctions. (A) *Xalas* (TREP1571) *vs Xalax* (TREP3344), (B) *Olivia* (TREP3219) vs. *Daniela* (TREP796). Lines indicate regions of sequence homology. (PDF 375 kb) Additional file 5: Distribution of distances (shown in nt) between the terminal monomer of the tandem array and the TE present at the TE/tandem array junction (*x-*axis). (A) TE/pSc200 junctions, (B) ТЕ/pSc250 junctions. The y-axis plots the ratio between the number of junctions harboring a given size of spacer DNA and the total number of junctions harboring the same TE. (PDF 341 kb) Additional file 6: Distribution of distances (shown in nt) between the first nucleotide of the TE and the closest full length TE (5′- or 3′-end) present at the TE/tandem array junction (*x-*axis). (A) TE/pSc200 junctions, (B) ТЕ/pSc250 junctions. The *y-*axis plots the ratio between the number of junctions harboring a given size of spacer DNA and the total number of junctions harboring the same TE. (PDF 416 kb) Additional file 7: DNA motif detection vs. TE occurrence in junctions “genomic DNA – tandem”. Example of the contingency table used to compute Fischer’s t-test for evaluation of the reads dataset containing junctions of tandem arrays with genomic DNA T-test checked the association between (a) detection of the certain DNA motif and (b) mapping of certain TE. Values *A, B, C, D* denote the numbers of reads. (DOC 25 kb)
