# Supplementary material for: The expansion of heterochromatin blocks in rye reflects the co-amplification of tandem repeats and adjacent transposable elements
Source: BMC Genomics. 2016 May 4;17:337. doi: 10.1186/s12864-016-2667-5 (PMC4857426; doi:10.1186/s12864-016-2667-5)
Supplement: Additional file 1: — Multimeric repeat units present in the central portion of the arrays. PFGE separation of BAC clones containing pSc119.2, TaiI and pSc200 arrays. Southern hybridizations probed with (left panel) pSc119.2 and (right panel) pSc200. Lane 1: BAC clone 84C15, lane 2: 130H7, lane 3: 230C21, lane 4: 230N4, lane 5: 241H2. The central panel illustrates the structure of the BAC clones. Black rectangles: non-array sequences. (PDF 90 kb) [file 12864_2016_2667_MOESM1_ESM.pdf]

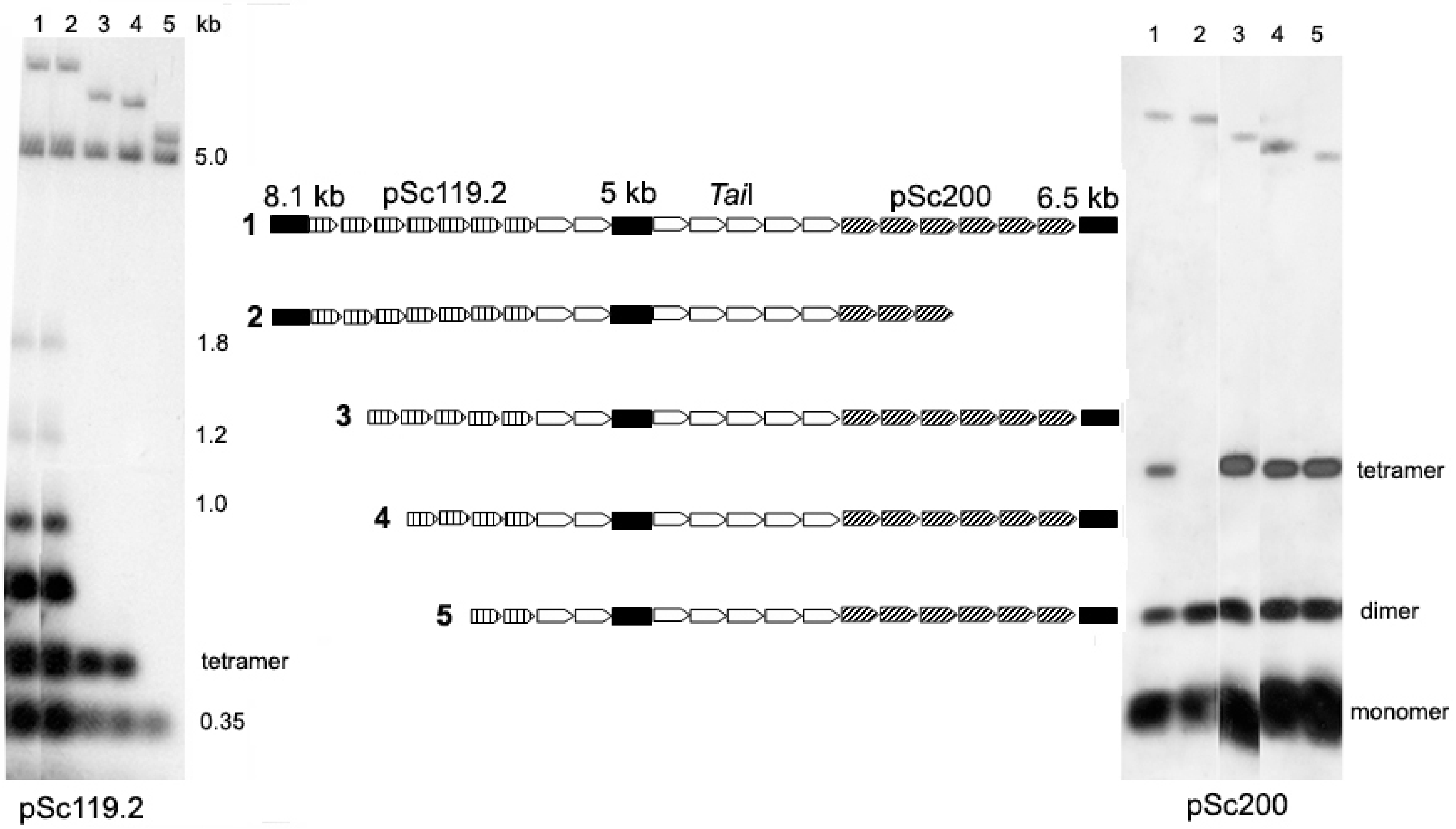

Additional file 1. Multimeric repeat units present in the central portion of the arrays.

PFGE separation of BAC clones containing pSc119.2, *Tai*I and pSc200 arrays. Southern hybridization probed with (left panel) pSc119.2 and (right panel) pSc200. Lane 1: BAC clone 84C15, lane 2: 130H7, lane 3: 230C21, lane 4: 230N4, lane 5: 241H2. The central panel illustrates the structure of the BAC clones. Black rectangles: non-array sequences.
