# Supplementary material for: The expansion of heterochromatin blocks in rye reflects the co-amplification of tandem repeats and adjacent transposable elements
Source: BMC Genomics. 2016 May 4;17:337. doi: 10.1186/s12864-016-2667-5 (PMC4857426; doi:10.1186/s12864-016-2667-5)
Supplement: Additional file 4: — Dot-plot alignment of TE sequences most highly enriched in the TE/tandem array junctions. (A) Xalas (TREP1571) vs Xalax (TREP3344), (B) Olivia (TREP3219) vs. Daniela (TREP796). Lines indicate regions of sequence homology. (PDF 375 kb) [file 12864_2016_2667_MOESM4_ESM.pdf]

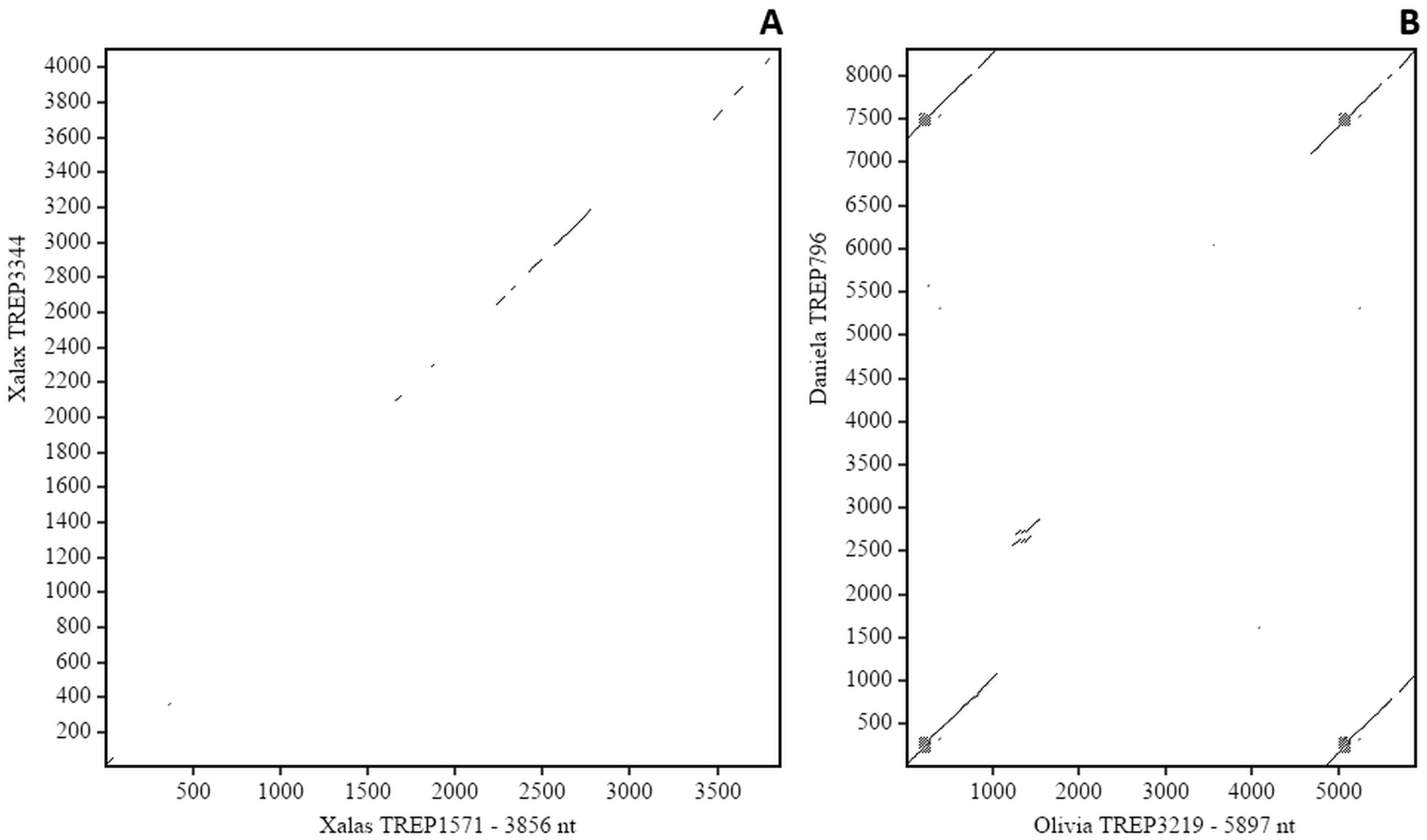

Additional file 2. Dot-plot alignment of TE sequences most highly enriched in the TE/tandem array junctions.

(A) *Xalas* (TREP1571) *vs Xalax* (TREP3344), **(B)** *Olivia* (TREP3219) *vs Daniela* (TREP796). Lines indicate regions of sequence homology.
