# Supplementary material for: The expansion of heterochromatin blocks in rye reflects the co-amplification of tandem repeats and adjacent transposable elements
Source: BMC Genomics. 2016 May 4;17:337. doi: 10.1186/s12864-016-2667-5 (PMC4857426; doi:10.1186/s12864-016-2667-5)
Supplement: Additional file 6: — Distribution of distances (shown in nt) between the first nucleotide of the TE and the closest full length TE (5′- or 3′-end) present at the TE/tandem array junction (x-axis). (A) TE/pSc200 junctions, (B) ТЕ/pSc250 junctions. The y-axis plots the ratio between the number of junctions harboring a given size of spacer DNA and the total number of junctions harboring the same TE. (PDF 416 kb) [file 12864_2016_2667_MOESM6_ESM.pdf]

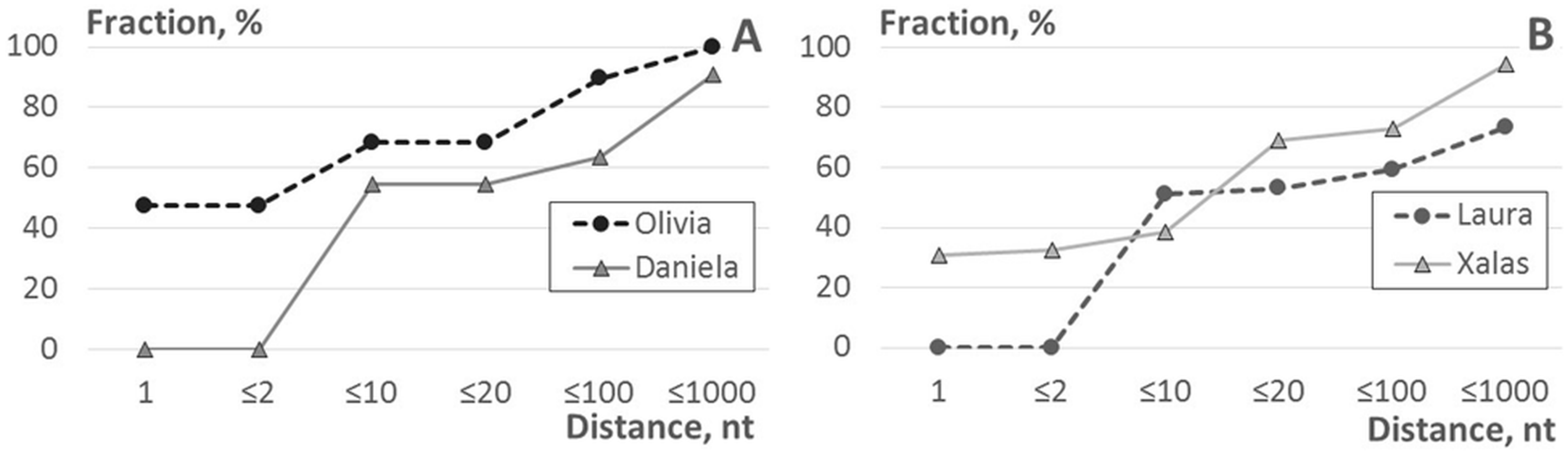

Additional file 4. Distribution of distances (shown in nt) between the first nucleotide of the TE and the closest full length TE (5'- or 3'-end) present at the TE/tandem array junction (*x*-axis). **(A)** TE/pSc200 junctions, **(B)** TE/pSc250 junctions. The *y*-axis plots the ratio between the number of junctions harboring a given size of spacer DNA and the total number of junctions harboring the same TE.
