# Supplementary material for: The expansion of heterochromatin blocks in rye reflects the co-amplification of tandem repeats and adjacent transposable elements
Source: BMC Genomics. 2016 May 4;17:337. doi: 10.1186/s12864-016-2667-5 (PMC4857426; doi:10.1186/s12864-016-2667-5)
Supplement: Additional file 7: — DNA motif detection vs. TE occurrence in junctions “genomic DNA – tandem”. Example of the contingency table used to compute Fischer’s t-test for evaluation of the reads dataset containing junctions of tandem arrays with genomic DNA T-test checked the association between (a) detection of the certain DNA motif and (b) mapping of certain TE. Values A, B, C, D denote the numbers of reads. (DOC 25 kb) [file 12864_2016_2667_MOESM7_ESM.doc]

**Additional file 5.** **DNA motif detection vs. TE occurrence in junctions “genomic DNA – tandem”**

Example of the contingency table used to compute Fischer’s t-test for evaluation of the reads dataset containing junctions of tandem arrays with genomic DNA T-test checked the association between (a) detection of the certain DNA motif and (b) mapping of certain TE. Values *A, B, C, D* denote the numbers of reads.

|  | | Number of reads | |
| --- | --- | --- | --- |
|  |  | All junctions | Junctions containing TEs |
| Motif | Present | *A* | *B* |
|  | Absent | *C* | *D* |
